# From competency mapping to curriculum development: Identifying gaps in an undergraduate general practice clerkship – a mixed-methods study

**DOI:** 10.3205/zma001873

**Published:** 2026-09-15

**Authors:** Dorothee Schüle, Benedikt Sonnek, Judith Lübbert, Sonia Kurczyk, Katharina Glassen, Svetla Loukanova

**Affiliations:** 1 Heidelberg University Hospital, Department of General Practice and Health Services Research, Heidelberg, Germany

**Keywords:** undergraduate medical education, competency-based education, general practice, family practice, clinical clerkship, curriculum, medical schools/organization & administration, teaching

## Abstract

**Objectives:**

This study aims to show the process of developing a competency-based medical education (CBME) General Practice (GP) block clerkship at the University of Heidelberg, aligned with frameworks such as CanMEDS, Entrustable Professional Activities (EPAs), and the National Competency-based Learning Objectives Catalogue (NKLM 2.0). Objectives are to identify competencies addressed in the current two-week clerkship, determine additional content needed to cover all required competencies, and assess student perspectives.

**Methods:**

A questionnaire completed by 165 students in 2023 evaluated perceived competence in various areas and their interest in GP before and after the clerkship. Data were analysed using a 5-point Likert scale ((1=fully agree to 5=completely disagree), and the curriculum was mapped to NKLM 2.0, CanMEDS, and EPAs to identify represented and missing competencies. Focus group meetings with lecturers and students were held to develop a new concept.

**Results:**

The current clerkship received an overall rating of 1.8 (SD: 1.06) but did not significantly increase interest in GP careers. Some competencies were well-covered, but areas like interprofessional skills or leadership and management were lacking. A six-week longitudinal clerkship was proposed to address these gaps, focusing on interprofessional collaboration, leadership, and patient-centred care, and including digital teaching, reflection and simulation training, and shadowing experiences.

**Conclusion:**

The proposed CBME curriculum addresses evolving needs in medical education. Successful implementation will require longitudinal integration, interdisciplinary cooperation, trained preceptors, and centralised coordination. This study provides a roadmap for emphasising key competencies, promoting GP career choice, and contributing to high-quality patient care.

## Zusammenfassung

**Zielsetzung:**

Diese Studie beschreibt den Prozess der Entwicklung eines kompetenzbasierten allgemeinmedizinischen Blockpraktikums an der Universität Heidelberg, das an Rahmenwerken wie CanMEDS, Entrustable Professional Activities (EPAs) und dem Nationalen Kompetenzbasierten Lernzielkatalog Medizin (NKLM 2.0) ausgerichtet ist. Ziel war es, die im bestehenden zweiwöchigen Blockpraktikum vermittelten Kompetenzen zu identifizieren, zusätzliche Inhalte zur Abdeckung aller erforderlichen Kompetenzen zu definieren sowie die Perspektive der Studierenden zu erfassen.

**Methoden:**

Im Jahr 2023 füllten 165 Studierende einen Fragebogen zur wahrgenommenen Kompetenzentwicklung und zum Interesse an der Allgemeinmedizin vor und nach dem Praktikum aus. Die Auswertung erfolgte mittels fünfstufiger Likert-Skala (1=stimme voll zu bis 5=stimme überhaupt nicht zu). Das Curriculum wurde anhand von NKLM 2.0, CanMEDS und EPAs kartiert, um vermittelte und fehlende Kompetenzen zu identifizieren. Fokusgruppen mit Studierenden und Lehrenden dienten der Entwicklung eines neuen Curriculummodells.

**Ergebnisse:**

Das bestehende Blockpraktikum erhielt eine Gesamtnote von 1,8 (SD: 1,06), führte jedoch nicht zu einer signifikanten Steigerung des Interesses an einer allgemeinmedizinischen Laufbahn. Einige Kompetenzen waren gut abgedeckt, während Bereiche wie interprofessionelle Zusammenarbeit sowie Führung und Management fehlten. Zur Schließung dieser Lücken wurde ein sechs Wochen umfassendes longitudinales Praktikum mit Schwerpunkt auf interprofessionelle Zusammenarbeit, Führungskompetenzen und patientenzentrierte Versorgung erarbeitet, welches digitale Lehrformate, Reflexions- und Simulationstrainings sowie Hospitationen integriert.

**Schlussfolgerung:**

Das vorgeschlagene kompetenzbasierte Curriculum trägt den sich wandelnden Anforderungen der medizinischen Ausbildung Rechnung. Für eine erfolgreiche Umsetzung bedarf es longitudinaler Integration, interdisziplinärer Zusammenarbeit, qualifizierter Lehrärztinnen und Lehrärzte sowie einer zentralen Koordination. Die vorliegende Studie bietet einen Leitfaden zur Stärkung zentraler Kompetenzen, zur Förderung der Berufswahl Allgemeinmedizin und zur Verbesserung der Versorgungsqualität.

## 1. Introduction

### 1.1. Concepts for competency-based medical education

The evolving field of medical education demands a shift in curricula from a traditional knowledge- and time-based approach to a competency-based medical education (CBME) [1]. Different frameworks regarding competencies as outcomes have been developed. In the German context, the National Competency-based Learning Objectives Catalogue for Medical Education (Nationaler Kompetenzbasierter Lernzielkatalog 2.0 - NKLM) as a national core curriculum provides guidance for medical faculties in developing competency-based programs ([https://www.nklm.de/], last access 08.04.2026). It integrates both CanMEDS (Canadian Medical Education Directives for Specialists) roles and Entrustable Professional Abilities (EPA), ensuring alignment with international competency models while addressing local regulatory requirements. The CanMEDS roles define graduates’ professional identity as Medical Expert, Scholar, Leader, Communicator, Collaborator, Health Advocate and Professional [2], while the concept of EPAs translates these roles into concrete, observable tasks that reflect real clinical responsibilities [3].Together, these three frameworks offer a multidimensional approach that complements and reinforces each other, supporting holistic professional development, allowing for practical assessment, and meeting both national standards and international best practices. 

Research highlights that GP clerkships substantially enhance competence development and foster positive attitudes towards the discipline [4], [5], [6], [7]. Competency-oriented clerkships and longitudinal curricula have also been shown to strengthen clinical skills and increase interest in general practice careers [8], [9], [10], [11].

### 1.2. Existing regulations and planned new structures for GP block clerkships 

Since 2013, the German Licensing Regulations for Doctors (Approbationsordnung für Ärzte, 2002, [https://www.gesetze-im-internet.de/_appro_2002/BJNR240500002.html], last access 08.04.2026) requires a two-week GP block clerkship. The medical education system in Germany is a six-year programme, with the first two years dedicated to fundamental scientific principles, the subsequent three years devoted to clinical science, and the final year allocated for a practical training period. The clerkship is incorporated into the clinical years. Placements in urban and rural primary care practices offer medical students opportunities to gain experience regarding common consultation issues, supported by theoretical training. Additionally, a 30-day unstructured elective in GP is required. At the University of Heidelberg, the integrated, modular, interdisciplinary medical education curriculum HeiCuMed provides the structure within which the GP block clerkship takes place [12]. While this reflects the traditional curricular structure, medical education in Germany is increasingly characterised by model study programmes and competency-oriented Z-curricula, in which preclinical and clinical content are interwoven, signalling an ongoing transformation towards integrated and longitudinally structured learning, e.g. at the Charité Berlin [13].

In 2017, a new competency-oriented licensing regulation for medicine in Germany was announced by policymakers [14]. They had envisioned a six-week longitudinal GP clerkship guided by NKLM 2.0 competencies, taking place throughout preclinical and clinical years. Predominantly primary care issues defined as strategies in long-term care of patients with chronic disease, handling multimorbidity, home visits, screening examinations, family medicine and nursing home care were to be focused on [15]. In anticipation of this restructuring of medical education, numerous projects aimed at improving the curriculum were put on hold. Subsequently, several years have passed, and the implementation of the so-called “Master Plan 2020” currently seems far off, or even on the verge of failing [16]. As a result, the initial announcement of a reform aimed at enhancing medical education, paradoxically, might have led to the opposite outcome. The ongoing postponement of the Master Plan 2020 is now perceived more as an obstacle rather than a driving force for new innovative teaching concepts. This highlights the need to integrate longitudinal, competency-based elements within the current regulations by expanding existing clerkships and improving coordination across semesters and teaching sites. Such approaches also make the model transferable to other faculties, regardless of future reforms.

### 1.3. Structure of current block clerkship at University of Heidelberg

The block clerkship includes eight on-site days in urban and rural practices, supported by preparatory online modules via an online tool (*El Medico*) using a flipped-classroom approach. Students apply knowledge in case-based workshops, complete logbook assignments during practice days, and conclude with a debriefing session focused on case presentations and professional reflection.

### 1.4. Aim of the study

In line with the frameworks above and the current regulatory context, this report describes the development of a competency-based GP block clerkship. The findings and reflections presented aim to inform and guide similar efforts to develop curricula in medical education elsewhere.

The problem addressed is to identify which of the competencies outlined by NKLM 2.0, CanMEDs, and EPAs are currently included in the existing two-week GP block clerkship, and to determine what additional teaching content should be incorporated in the clerkship to ensure comprehensive coverage of all required competencies. 

The aim of this report was to guide further development of a competency-based GP clerkship through student evaluation and mapping of competencies. How does the current two-week GP block clerkship perform in terms of perceived competence development? What gaps do students and curriculum mapping reveal that must be addressed to better align the clerkship with competency-based frameworks and include considerations for a future longitudinal model? 

## 2. Project description

First, to evaluate the quality of the current GP block clerkship, a questionnaire consisting of 26 closed questions was used (see attachment 1 ). In addition to assessing interest in GP before and after the clerkship, it evaluates acquirement of specific general medical competencies and done activities (e.g., administering vaccinations) as well as the quality of teaching in the respective teaching practices. The assessment is carried out using a 5-point Likert scale (1=fully agree to 5=completely disagree). Of all students participating in the block clerkship in winter of 2023/2024 (n=221), 165 completed the questionnaire (74,66%). There was no financial remuneration. The data were analysed using the descriptive statistical tool integrated in the questionnaire. 

For the initial focus group, three volunteer students from our pool of preclinical teaching tutors participated – two with prior experience in the GP block clerkship and one without. They were introduced to the relevant competency frameworks (NKLM 2.0, CanMEDS, EPAs) and the possible legislative changes affecting clerkship structure. The group offered feedback on the current clerkship and contributed ideas for the future model, addressing both organisational aspects and curricular content. The written focus group protocol was subsequently analysed using a deductive thematic analysis approach, guided by the predefined competency frameworks (NKLM 2.0, CanMEDS, and EPAs). As no verbatim transcripts were produced, the meeting notes served as the primary data source. Two researchers (JL and DS) jointly reviewed the protocol, coded relevant statements according to the established thematic domains, and collaboratively developed overarching themes. This consensual process enhanced analytical transparency and ensured consistency in the interpretation of findings.

Second, to assess which competencies are already covered by the current block clerkship, all described teaching materials – including lecture slides, online modules in El Medico, and the logbook – were reviewed by JL and DS. These materials were systematically mapped to the relevant competency domains/roles/EPAs, using the CanMEDs framework, NKLM 2.0 core competencies and EPAs (see table 1 (Tab. 1)). Where applicable, each teaching element was linked to a corresponding EPA, NKLM 2.0 core competency, and CanMEDs role. Competencies and roles that were absent or only marginally represented were identified. The mapping results, the outcomes from the first focus group, as well as the relevant competency frameworks (NKLM 2.0, CanMEDS, EPAs) and the possible legislative changes affecting clerkship structure, were then presented by JL and DS to a second focus group comprising 11 members of the GP teaching staff, most of them involved in teaching in the GP clerkship. Those provided feedback and collaboratively contributed to the design of the future clerkship model. A comprehensive protocol documented the focus group discussion, which was subsequently examined through deductive thematic analysis in the same form as the first focus group protocol by JL and DS to identify key priorities and structural elements for the revised longitudinal clerkship model. For peer debriefing, results were provided to all members of the second focus group via email and feedback was incorporated.

Based on the following competency catalogues, a new clerkship concept was subsequently developed, involving all participants, with a detailed specification of all competencies that are to be taught within the framework of the block clerkship. 

### 2.1. CanMEDs

The CanMEDS roles framework is a comprehensive model used globally in medical education to define the competencies required of physicians to deliver high-quality, patient-centred care within increasingly complex healthcare environments. Developed by the Royal College of Physicians and Surgeons of Canada, CanMEDS identifies seven key roles that physicians must embody to meet the needs of their patients (see table 2 (Tab. 2)).

### 2.2. EPAs

EPAs are key tasks or responsibilities in clinical practice that trainees must be able to perform independently once they have acquired the necessary competencies. EPAs bridge the gap between competencies and real-world practice, offering a practical way to assess readiness for unsupervised clinical duties [3], [17]. Originally developed for graduate medical education, EPAs have been adapted to undergraduate medical education as professional activities “every resident should be able to do without direct supervision on day one of residency, regardless of specialty” [18]. The degree of supervision decreases throughout the process of mastering an EPA - from observation to direct supervision, then to reactive and indirect supervision [17], [19].

Several propositions of core EPAs for graduates have been made. In this report, it was chosen to modify those adapted to the German context by Holzhausen et al. [20], extended to include two additional EPAs included in the NKLM 2.0 (see table 3 (Tab. 3)).

### 2.3. National Competency Based Learning Objectives Catalogue for Medicine (NKLM 2.0)

The NKLM was first introduced in 2015 to improve the quality of medical education across Germany. It was developed through extensive collaboration among the Medical Faculties Convention (MFT) and the Association for Medical Education (GMA), together with representatives of government bodies, professional societies and medical students. Feedback from the initial version led to the refined NKLM 2.0, which was published in 2021, with further versions under development. The competency-based framework outlines the essential competencies that students must acquire during medical education. For each learning objective, four competency levels are defined – from factual knowledge to the ability to act independently.

The NKLM 2.0 consists of lists of diseases, reasons for consultation, a graduate profile based on EPAs, disease-related learning objectives and defines seven core competency domains (see table 4 (Tab. 4)).

## 3. Results

### 3.1. Results of the evaluation

In 2023, a total of 221 students participated in the GP block clerkship, 165 completed the questionnaire. The clerkship received an overall rating of 1.8 (SD: 1.06). The interest in pursuing a career in GP was not significantly increased by the block clerkship (Before the clerkship: 3.16 [SD: 1.09], after the clerkship: 2.68 [SD: 1.13], *p*=0.122). In the assessment of the queried competencies (see attachment 1 ), students reported a high perceived competence with an average of 1.83 (SD: 1.02). The competencies evaluated include: conducting GP decision-making, presenting cases using the SOAP format for both an acutely ill patient and a chronically ill patient, performing a home visit under supervision, and managing a transition patient (transfer from inpatient to outpatient care or vice versa).

### 3.2. Represented competencies

From a curricular and teaching perspective, the following competencies are represented in the current GP block clerkship. They are taught through e-learning sessions, workshops before and after the on-site days, and assignments that students must complete to pass. Table 1 (Tab. 1) gives an overview of the competencies, represented in the GP block clerkship, according to the NKLM 2.0, CanMEDs and EPAs. 

### 3.3. Missing competencies and topics

As shown in table 1 (Tab. 1), the competency domains and professional roles of leadership and management/leader as well as interprofessional competencies/collaborator are not represented in the current curriculum of the block clerkship. The competency domains and professional roles of medical scientific skills/scholar and professional conduct, ethics, medical history and law/professional are only marginally covered. EPAs 4.4 (management of a ward or doctor’s office), 5.1 (recognise an emergency situation and act upon it) and 5.3 (contribute to patient safety and system improvement) are not part of the current curriculum. However, some of those competencies are considered in other parts of the GP curriculum, but either in form of an optional eLearning course in the preclinical teaching (interprofessional care) or as part of the elective track “primary care”, which is not for all students, or as part of the informal teaching in some GP practice placements, depending on their size, network, etc. 

### 3.4. Concept for a longitudinal block clerkship

Including the missing competencies and EPAs, as well as focus thematic areas like planetary health, digital transformation, patient safety and the predominantly primary care issues defined by licensing regulation proposals (strategies in long-term care of patients with chronic disease, handling multimorbidity, home visits, screening examinations, family medicine and nursing home care), the following concept for an extended GP block clerkship was developed. Students’ perspective using evaluation data was considered. 

Rather than representing a fully predefined longitudinal curriculum, the proposed model outlines a structured framework for the progressive integration of competency-based learning elements over an extended clerkship period. The framework (see figure 1 (Fig. 1)) illustrates the interaction between thematic areas, competency domains, and teaching formats.

High-quality patient-centred care was defined as the central goal of the new holistic framework. The CanMEDs roles as competency domains build the foundation of the curriculum and are acquired through and reflected on in the different formats and thematic areas. Predominantly primary care issues are processed through practice-based learning and preparatory teaching, enhanced and complimented by digital online teaching on theoretical concepts. The digital teaching enables asynchronous learning, e.g., during students’ commute to on-site clerkship days with practical assignments to complete during the day. A reflection and simulation training focuses on professional attitude, communication, scientific research and evidence-based medicine. Interprofessional collaboration is emphasised by way of a shadowing experience in students’ field of choice. Continuous re-evaluation helps faculty in further development of the curriculum, whereas feedback from preceptors and foundation on competency-based curricula as outlined in the logbook supports students in assessing their learning progress. 

Attachment 2 provides a structured overview of thematic areas and possible teaching formats in alignment with the underlying competency frameworks. This overview is not intended as a fixed curriculum schedule, but rather as a modular framework that supports local curriculum development and adaptation depending on institutional resources and priorities. While lectures are listed as one possible format, they are intended to be complemented or replaced by interactive and case-based approaches where feasible. Attachment 3 provides an example of how progressive competence development could be operationalised in a longitudinal clerkship.

Regarding transferability, it can be said that core elements include on-site placements in GP, competency-based supervision, and structured reflection, whereas specific teaching formats (e.g., lectures, online modules, simulation) can be adapted depending on local resources. Attachment 4 contains a newly developed competency-based logbook outlining specific competencies and associated learning objectives, classified into obligatory and optional learning objectives, ready to implement at other locations. 

A more detailed week-by-week structure was beyond the scope of this report and will be subject of future implementation work.

## 4. Discussion

Several model curricula in Germany have introduced longitudinal and competency-based elements, including early and continuous exposure to primary care (e.g., Charité Berlin [21], Witten/Herdecke [22], Marburg [23]). However, these approaches are typically embedded within broader curricular structures and vary considerably in their implementation. Clearly structured and systematically longitudinal GP curricula remain limited, particularly regarding systematic approaches to identifying and addressing competency gaps. 

Against this background, the present study can provide a pathway for further development of GP clerkships through competency mapping and proposition of a curriculum framework.

### 4.1. What is needed for implementation?

#### 4.1.1. Longitudinal curriculum 

Park et al. [7] demonstrated in a systematic review that clinical skills can be learned just as effectively, if not better, in GP settings. It was shown that early clinical placements can help motivate students, and that GP serves as a socio-cultural learning space for professionalism and interprofessional collaboration. Several studies have shown that earlier, longer, and longitudinal learning experiences in primary care even promote a GP career choice [9], [24], [25]. The German Society of General Practice also call for longitudinal embedding throughout the whole of medical school from the preclinical phase to the practical year to reflect the breadth of the subject and continuity of care [26].

In order to implement the proposed changes, GP needs greater prioritisation and more course time. Although legislative reform appears unlikely soon, longitudinal integration can still be achieved within current regulations. Cross-semester coordination with other disciplines has the potential to facilitate interdisciplinary teaching of aspects over the course of undergraduate training that are not specific to general practice, for example, communication with limitations or error management. Preclinical integration, as modelled at the University of Heidelberg through a teaching programme focusing on history-taking and physical examination, overseen by the GP department and incorporating on-site GP clerkship days, can be conceptualised as part of a longitudinal curriculum. This integration then is facilitated through the utilisation of the frameworks previously outlined. It is imperative that particular options are assessed on a site-specific basis.

#### 4.1.2. Interdisciplinary cooperation within faculty

CBME concepts and regulations apply to all preclinical and clinical courses. Coordination between disciplines is necessary to avoid repetition or focusing on certain skills while others are taught little or not at all. Longitudinal competence tracks, such as those already in place at several universities for communication skills [27], [28], can map existing programmes for the overarching competence domains and identify gaps.

#### 4.1.3. Teaching preceptors (train the trainer) 

Curriculum changes and the introduction of the competency-based curriculum need to be communicated to preceptors, as the majority of teaching in GP is decentralised and takes place in teaching practices. A Train-the-Trainer (TTT) curriculum is essential for enhancing the teaching abilities of GPs. Studies have shown that these programmes significantly improve teaching skills, confidence, and attitudes toward teaching in clinical settings [29], [30]. TTT including feedback, motivation and question framing is already implemented at Heidelberg University and can be updated and expanded. In the absence of such a programme, one should be developed. 

#### 4.1.4. Coordination by institute of general practice

The establishment of a coordination centre at the institute of general practice is important for the development of a longitudinal, competency-based GP curriculum. It ensures effective communication between university departments, teaching practices, students and regulatory bodies, while simultaneously integrating the latest scientific findings. Such a centralised coordination serves to enhance curriculum quality, ensures legal compliance, and aligns clinical training with educational goals as has been shown for postgraduate training [31].

### 4.2. Real life check – what can be done under current circumstances?

Given that there is currently no prospect for changing the existing framework conditions, ways should be found to integrate the concept of CBME into the current medical education system. One example could be the implementation of a competency-based logbook for the block clerkship, as shown in attachment 4 [4]. In addition to a significant increase in competencies, positive experiences have also led to enhanced motivation for future work in GP [5], [32]. For the successful implementation of such a logbook, close collaboration with and guidance from teaching physicians in a TTT model is essential and beneficial. 

Another example might be the development of additional online training sessions covering currently missing competencies and topics as optional learning opportunities. At University of Heidelberg this intent is supported by new ongoing projects for longitudinal integration of cross-cutting subjects in medical education, including: professional identity formation, patient safety, interdisciplinary patient care, scientific skills, and digitalisation. In addition, the teaching practice training programme is being further developed, updated and standardised to strengthen didactic skills and incorporate the above areas. 

To work resource-efficiently and identify gaps in the coverage of existing competency catalogues by current teaching concepts, the curricula of university locations should be thoroughly examined within the framework of interdisciplinary collaboration among various departments. Potential gaps should be addressed through new teaching and training concepts. The proposed measures should be followed by a feasibility study to evaluate whether and to what extent the competency-based learning objectives outlined in the new curriculum can be implemented under the current structural and organisational conditions. This study would assess the practical feasibility, acceptance, and resource requirements of the longitudinal clerkship model after its initial implementation.

### 4.3. Strengths and limitations

This project has several strengths and limitations. One limitation is that the focus group of teaching physicians consisted mainly of staff members from the Department of General Practice. This may have introduced institutional bias and reduced the diversity of viewpoints. Likewise, the participation of student tutors in the first focus group may have favoured more motivated students. As no verbatim transcripts were produced, relying on written protocols could have limited the analytical depth of the thematic analysis. The evaluation data were taken from the standard institutional questionnaire, which allows only a limited number of items to maintain a high response rate.

Despite these limitations, the project’s strengths include a systematic mapping of competencies against established frameworks (NKLM 2.0, CanMEDS, EPAs), a participatory development process involving both students and teachers, and a pragmatic design that enables transfer to other medical faculties.

## 5. Conclusion

The development of a competency-based GP block clerkship at the University of Heidelberg addresses the evolving needs in medical education, aligning with national and international frameworks and standards. The current clerkship covers several key competencies but lacks comprehensive representation of all NKLM 2.0 overarching domains, CanMEDs roles and EPAs. The proposed pathway to develop a longitudinal clerkship aims to fill these gaps, emphasising interprofessional collaboration, leadership, and patient-centred care. Successful implementation will require longitudinal integration, interdisciplinary cooperation, trained preceptors, and centralised coordination and evaluation. While legislative uncertainty remains, the outlined strategy offers a practical roadmap for enhancing GP education under current circumstances, ultimately contributing to high-quality patient care, promoting GP career choice and potentially inspiring similar efforts worldwide.

## Notes

### Use of artificial intelligence

ChatGPT (OpenAI, GPT-5.3) was used to support drafting and structuring parts of the manuscript (approximately 15-20% of the text, 18 prompts). All AI-generated content was critically reviewed and revised by the authors, who take full responsibility for the final manuscript. 

### Authors’ ORCIDs

- Dorothee Schüle: [0009-0007-2337-2287]
- Benedikt Sonnek: [0000-0003-1055-6611]
- Judith Lübbert: [0009-0002-3803-0777]
- Katharina Glassen: [0000-0002-8892-8625]
- Svetla Loukanova: [0000-0003-3183-414X]

## Competing interests

The authors declare that they have no competing interests. 

## Supplementary Material

Evaluation questionnaire for the general practice block clerkship

Longitudinal integrated primary care block clerkship framework – specifications

Example of longitudinal model

Competency-based logbook for general practice block clerkship

## Figures and Tables

**Table 1:**
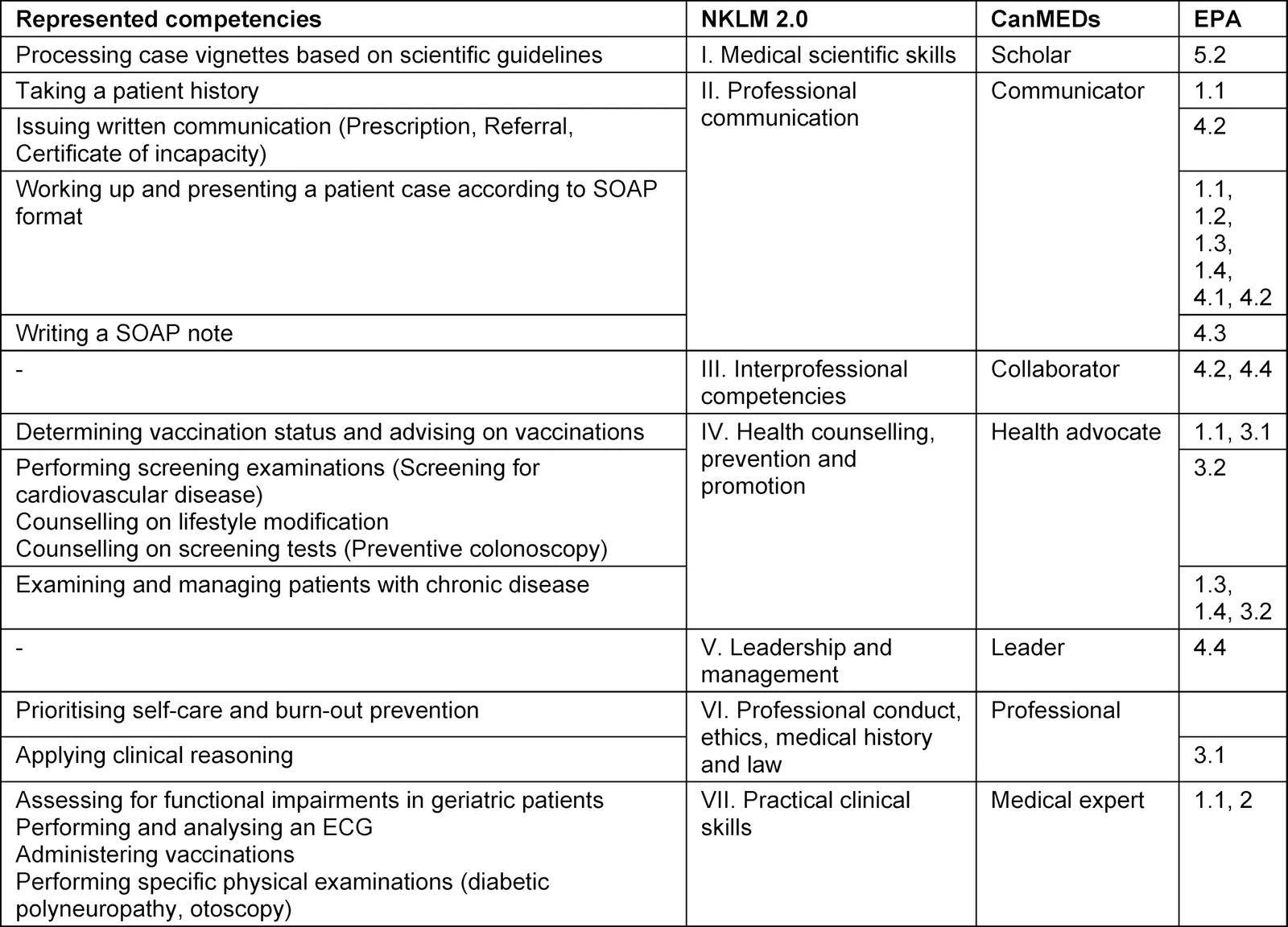
Mapping of competencies represented in the current GP block clerkship with NKLM 2.0, CanMEDs and EPAs

**Table 2:**
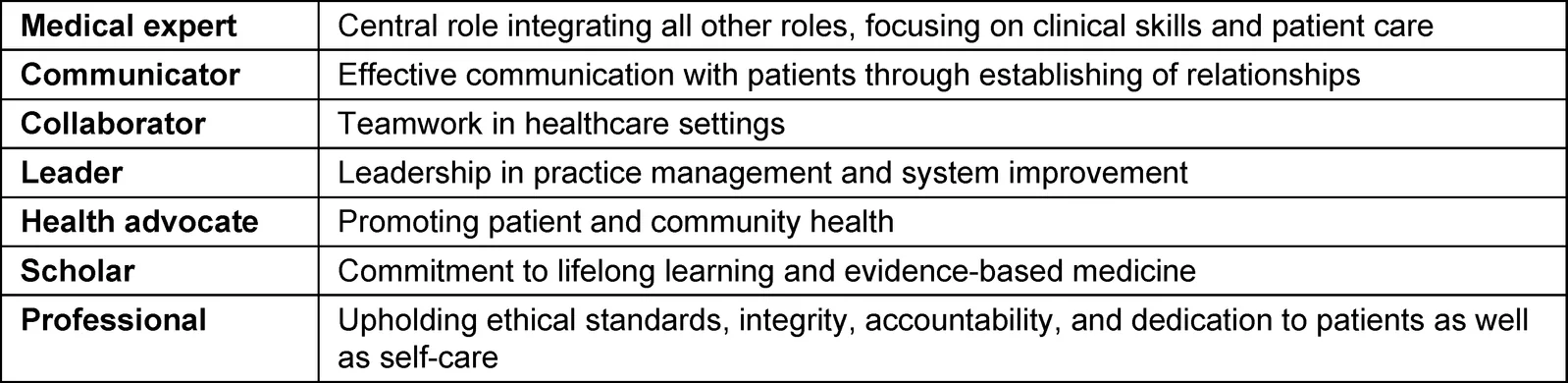
CanMEDS roles [2]

**Table 3:**
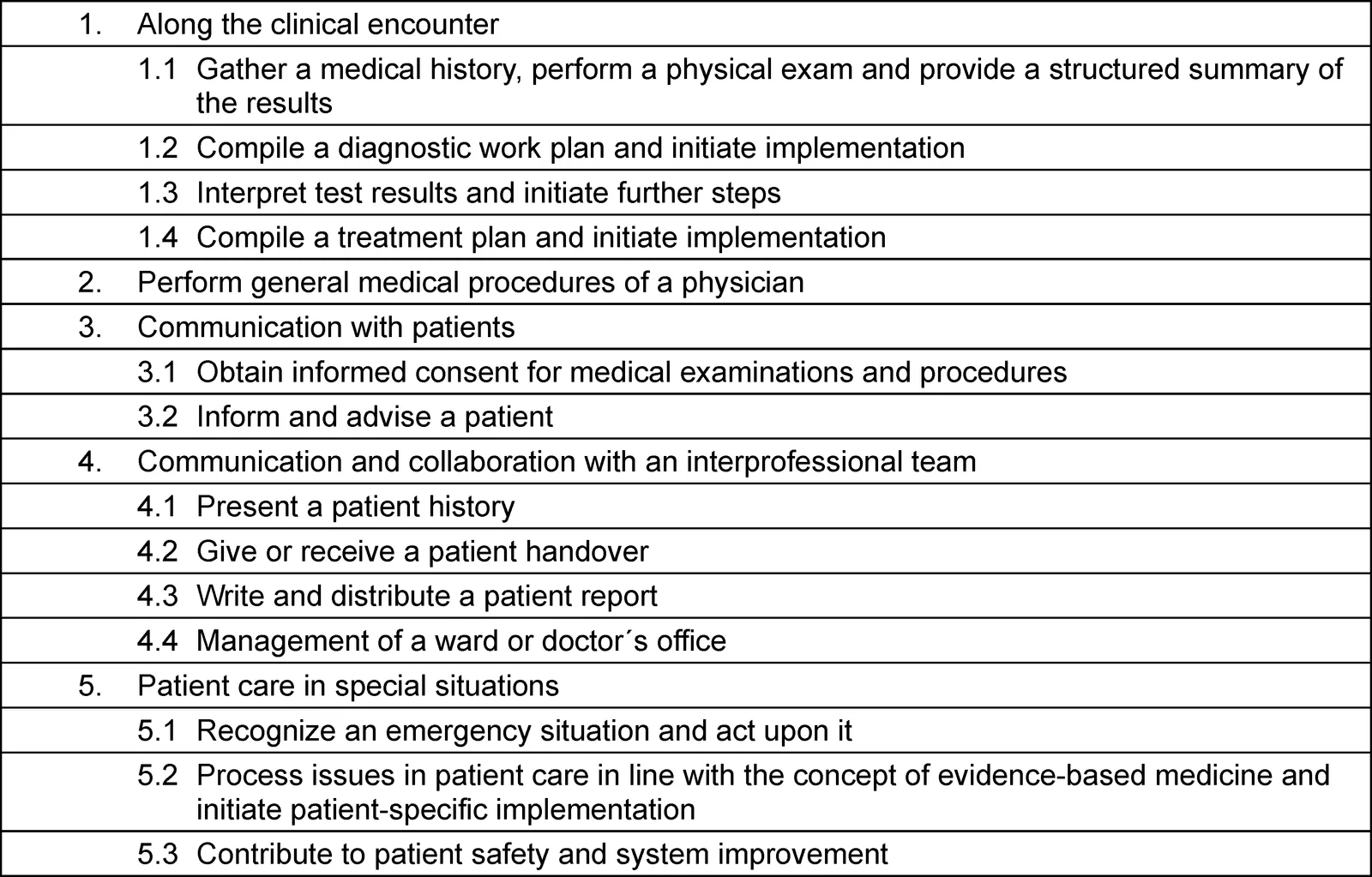
List of EPAs, modified according to [20] and NKLM 2.0

**Table 4:**
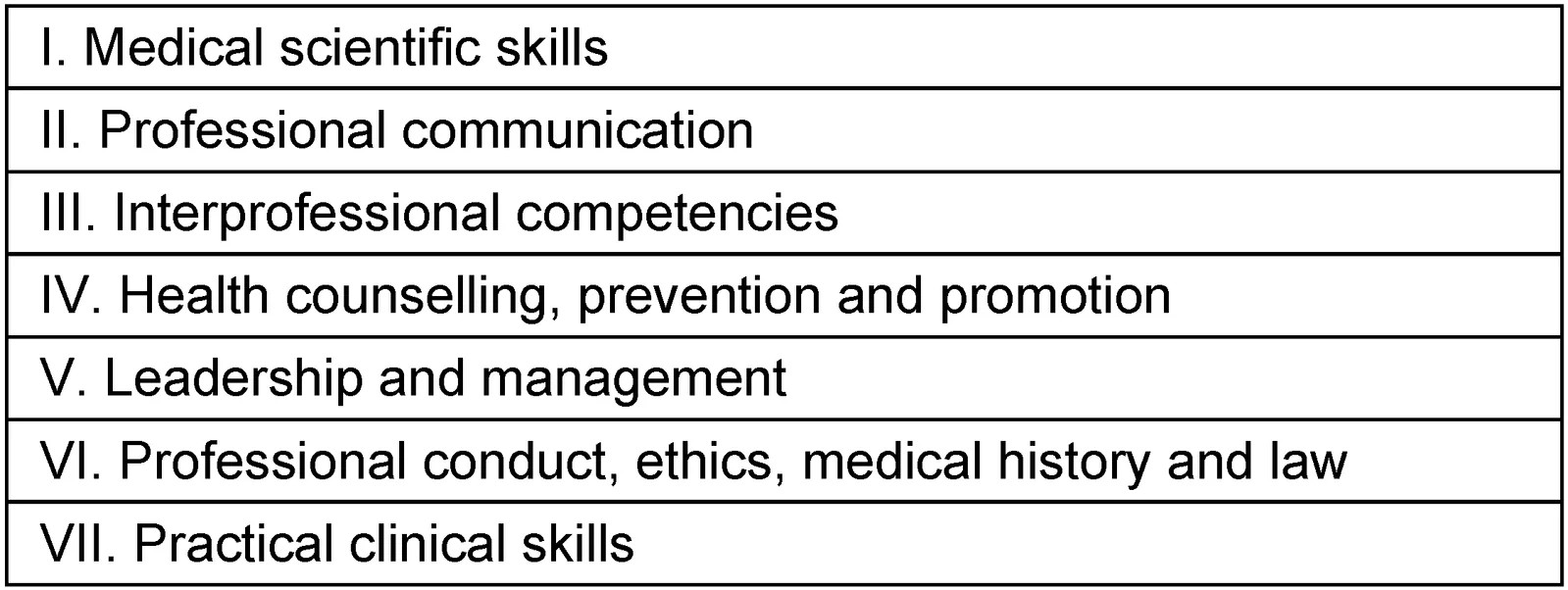
Core competency domains in NKLM 2.0

**Figure 1:**
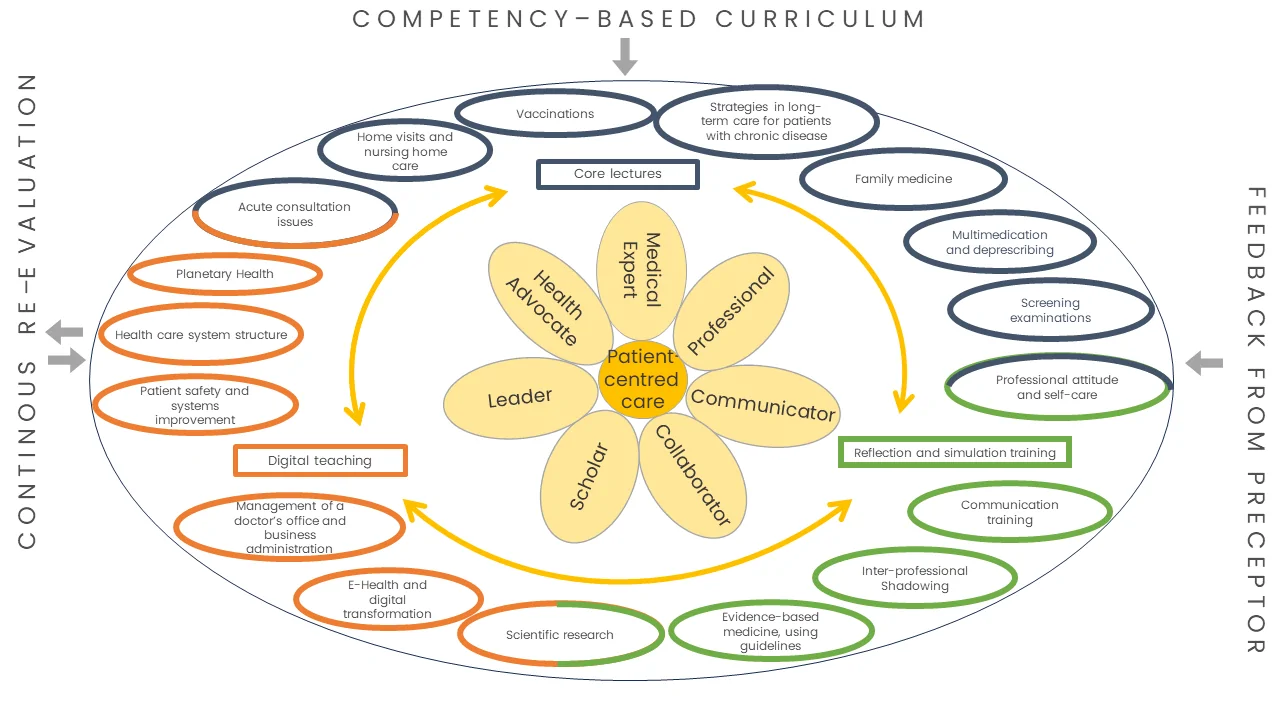
Longitudinal integrated primary care block clerkship framework
