# Supplementary material for: From competency mapping to curriculum development: Identifying gaps in an undergraduate general practice clerkship – a mixed-methods study
Source: GMS J Med Educ. 2026 Sep 15;43(6):Doc79. doi: 10.3205/zma001873 (PMC13617322; doi:10.3205/zma001873)
Supplement: Supplementary file 1 [file JME-43-79-s-001.pdf]

Attachment 1: Evaluation questionnaire for the general practice block clerkship

1. Interest

1.1. My interest in general practice prior to the block clerkship was:
o Very high o High o Indifferent o Low o Very Low

1.2. My interest in general practice is now:
o Very high o High o Indifferent o Low o Very Low

2. Activities

I was able to perform the following activities by myself under the supervision of the preceptor:

2.1 Issue a prescription (public or private health insurance)
o Completely applies o Applies o Indifferent o Partially applies o Does not apply

2.2 Assess incapacity for work and issue a sick note
o Completely applies o Applies o Indifferent o Partially applies o Does not apply

2.3 Create vaccination status and administer vaccination
o Completely applies o Applies o Indifferent o Partially applies o Does not apply

2.4 Screening examination with risk counselling
o Completely applies o Applies o Indifferent o Partially applies o Does not apply

2.5 Basic geriatric assessment (e.g., Barthel Index, Timed Up & Go, Tandem Stand, etc.)
o Completely applies o Applies o Indifferent o Partially applies o Does not apply

2.6 Disease Management Programme (DMP) examination and documentation for Type 2 Diabetes Mellitus
o Completely applies o Applies o Indifferent o Partially applies o Does not apply

2.7 Otoscopy
o Completely applies o Applies o Indifferent o Partially applies o Does not apply

2.8 Perform at least two ECGs, assess the findings, and then discuss the results with preceptor
o Completely applies o Applies o Indifferent o Partially applies o Does not apply

2.9 Provide counselling on colonoscopy or iFOBT as part of cancer screening
o Completely applies o Applies o Indifferent o Partially applies o Does not apply

Attachment 1 to Schüle D, Sonnek B, Lübbert J, Kurcrzyk S, Glassen K, Loukanova S. *From competency mapping to curriculum development: identifying gaps in an undergraduate general practice clerkship – a mixed-methods study*. GMS J Med Educ. 2026;43(6):Doc79. DOI: 10.3205/zma001873

3. Competencies

I feel confident …

3.1 To describe the process of general practice decision making (watchful waiting approach / exclusion of potentially dangerous courses of illness)
o Completely applies o Applies o Indifferent o Partially applies o Does not apply

3.2 To present a case using SOAP format: newly diagnosed patient
o Completely applies o Applies o Indifferent o Partially applies o Does not apply

3.3 To present a case using SOAP format: patient with a chronic illness or patient at the interface of different care settings
o Completely applies o Applies o Indifferent o Partially applies o Does not apply

3.4 To present a case using SOAP format: patient during follow-up
o Completely applies o Applies o Indifferent o Partially applies o Does not apply

3.5 To present a case using SOAP format: patient during home visit
o Completely applies o Applies o Indifferent o Partially applies o Does not apply

4. Learning opportunities

4.1. My preceptor defined learning objectives and learning opportunities with me at the beginning of the block clerkship
o Completely applies o Applies o Indifferent o Partially applies o Does not apply

4.2. I was able to accompany my preceptor in all medical activities in the practice and during home visits
o Completely applies o Applies o Indifferent o Partially applies o Does not apply

4.3. I felt well integrated into the practice team
o Completely applies o Applies o Indifferent o Partially applies o Does not apply

4.4. I found it easy to discuss my questions and concerns with the preceptor
o Completely applies o Applies o Indifferent o Partially applies o Does not apply

4.5. I was able to contribute my interests and skills
o Completely applies o Applies o Indifferent o Partially applies o Does not apply

4.6. I received regular specific feedback on my practical skills
o Completely applies o Applies o Indifferent o Partially applies o Does not apply

4.7. I received regular specific feedback on my behaviour towards patients
o Completely applies o Applies o Indifferent o Partially applies o Does not apply

4.8. My supervising doctor had a final discussion with me, providing mutual feedback
o Completely applies o Applies o Indifferent o Partially applies o Does not apply

4.9. In retrospect, I feel that the introductory event adequately prepared me for the block clerkship
o Completely applies o Applies o Indifferent o Partially applies o Does not apply

4.10. I rate my block clerkship in the teaching practice with the school grade
o 1- Very good o 2- good o 3 – satisfactory o 4 – sufficient o 5 – inadequate o 6 - insufficient

Attachment 1 to Schüle D, Sonnek B, Lübbert J, Kurczyk S, Glassen K, Loukanova S. *From competency mapping to curriculum development: identifying gaps in an undergraduate general practice clerkship – a mixed-methods study*. GMS J Med Educ. 2026;43(6):Doc79. DOI: 10.3205/zma001873
