# Supplementary material for: From competency mapping to curriculum development: Identifying gaps in an undergraduate general practice clerkship – a mixed-methods study
Source: GMS J Med Educ. 2026 Sep 15;43(6):Doc79. doi: 10.3205/zma001873 (PMC13617322; doi:10.3205/zma001873)
Supplement: Supplementary file 2 [file JME-43-79-s-002.pdf]

Attachment 2: Longitudinal integrated primary care block clerkship framework – specifications

	Thematic areas	Competency training	CanMEDs	NKLM 2.0	EPA
**Core lectures about predominantly primary care issues**	Acute consultation issues	Lectures about most common reasons for encounter	Medical expert	VII. Practical clinical skills	1.1<br>1.2<br>1.3<br>1.4
	Home visits and nursing home care	Lecture/Online course	Collaborator,<br>Medical expert	III. Interprofessional competencies,<br>VII. Practical clinical skills	1.1<br>1.2<br>1.3<br>1.4<br>4.2
	Vaccinations	Online course, training video and case vignettes determining vaccination status and advising on vaccinations	Health advocate,<br>Medical Expert	IV. Health counselling, prevention and promotion,<br>VII. Practical clinical skills	1.4 2<br>3.1
	Strategies in long-term care for patients with chronic disease	Longitudinal care for patient with chronic illness, alternatively standardised patient story, lecture and case vignettes	Communicator,<br>Collaborator,<br>Health advocate	II. Professional communication,<br>III. Interprofessional competencies,<br>IV. Health counselling, prevention and promotion	1.4<br>3.2<br>4.2<br>5.3
	Family medicine	Lecture	Communicator,<br>Health advocate,<br>Medical expert	II. Professional communication,<br>IV. Health counselling, prevention and promotion,<br>VII. Practical clinical skills	2<br>3.2
	Multimedication and deprescribing	Lecture and case vignettes	Health advocate	IV. Health counselling, prevention and promotion	1.4<br>3.2
	Screening examinations	Lecture and training video	Communicator,<br>Health advocate	II. Professional communication,<br>IV. Health counselling, prevention and promotion	1.1 2<br>3.2
	Professional attitude and self-care	Reflection in group seminars (e.g. dealing with embarassment in exam situations, burn-out prevention and self-care)	Professional	VI. Professional conduct, ethics, medical history and law	
**Digital teaching**	Acute consultation issues	Processing case vignettes for common reasons of encounter based on scientific guidelines	Medical expert,<br>Scholar	I. Medical scientific skills,<br>VII. Practical clinical skills	1.1<br>1.2<br>1.3<br>1.4<br>5.2

Attachment 2 to Schüle D, Sonnek B, Lübbert J, Kurcrzyk S, Glassen K, Loukanova S. *From competency mapping to curriculum development: identifying gaps in an undergraduate general practice clerkship – a mixed-methods study*. GMS J Med Educ. 2026;43(6):Doc79. DOI: 10.3205/zma001873

	Planetary Health	Online course	Leader	V. Leadership and management	5.3
	Healthcare system structure	Online course	Leader	V. Leadership and management	5.3
	Patient safety and systems improvement	Quality management system contribution in the form of a standardised sheet (SOP, patient information sheet, questionnaire, etc.)	Leader	V. Leadership and management	4.4<br>5.3
	Management of a doctor's office and business administration	Simulation game	Leader,<br>Collaborator	V. Leadership and management,<br>III. Interprofessional competencies	4.4
	E-health and digital transformation	Online course	Leader	V. Leadership and management	5.3
	Scientific research	Processing case vignettes for common reasons of encounter based on scientific guidelines	Scholar, Medical Expert	I. Medical scientific skills, VII. Practical Clinical Skills	1.1<br>1.2<br>1.3<br>1.4<br>5.2
**Reflection and simulation training**	Scientific research	EVIDENZ project (Leading a study about doctor-patient-encounter with subsequent qualitative and quantitative analysis)	Scholar	I. Medical scientific skills	
	Evidence-based medicine, using guidelines	Processing patient cases for common reasons of encounter based on scientific guidelines	Scholar,<br>Medical expert	I. Medical scientific skills,<br>VII. Practical clinical skills	1.1<br>1.2<br>1.3<br>1.4<br>5.2
	Interprofessional shadowing	Work shadowing at 1-2 other places (pharmacy, podiatry, physiotherapy, home nursing team, (addiction) counselling centre, occupational therapy, speech therapy, employment agency, rehabilitation centre etc.)	Collaborator	III. Interprofessional competencies	4.1<br>4.2<br>4.3<br>4.4
	Communication training	Interprofessional workshop, Training in history taking, Dealing with limitiations in communication	Communicator,<br>Collaborator	II. Professional communication,<br>III. Interprofessional competencies	3.1<br>3.2<br>4.1<br>4.2<br>4.3<br>4.4
	Professional attitude and self-care	Small group workshop	Professional	VI. Professional conduct, ethics, medical history and law	
