# Supplementary material for: From competency mapping to curriculum development: Identifying gaps in an undergraduate general practice clerkship – a mixed-methods study
Source: GMS J Med Educ. 2026 Sep 15;43(6):Doc79. doi: 10.3205/zma001873 (PMC13617322; doi:10.3205/zma001873)
Supplement: Supplementary file 3 [file JME-43-79-s-003.pdf]

Attachment 3: Example of longitudinal model

**Point in time**		**Week 1**	**Week 2**	**Week 3 & 4**	**Week 5 & 6**	
		**Preclinical training (2nd semester)**	**Preclinical training (4th semester)**	**Beginning of clinical training**	**End of clinical training**	**Over course of longitudinal clerkship**
**CanMEDs**	**NKLM 2.0**					
Scholar	I. Medical scientific skills		EVIDENZ project (Leading a study about doctor-patient-encounter with subsequent qualitative and quantitative analysis)	Processing case vignettes for common reasons of encounter based on scientific guidelines	Processing case vignettes for common reasons of encounter based on scientific guidelines	
Communicator	II. Professional communication	History taking	Written communication/forms, encouraging salutogenesis	Discussing therapy, Shared Decision Making	Dealing with limitations in communication (e.g. language barrier, impaired hearing)	Longitudinal care for patient with chronic illness, alternatively standardised patient story
Collaborator	III. Inter-professional competencies		Interprofessional communication	Management of a doctor's office		Work shadowing at 1-2 other places (pharmacy, podiatry, physiotherapy, home nursing team, (addiction) counselling centre, occupational therapy, speech therapy, employment agency, rehabilitation centre etc.)
Health advocate	IV. Health counselling, prevention and promotion	Vaccinations	Screening examinations	Disease management programs	Multimedication and deprescribing	Longitudinal care for patient with chronic illness, alternatively standardised patient story
Leader	V. Leadership and management	Healthcare system structure	Planetary health	eHealth and digital transformation, simulation game: management of a doctor's office and business administration		Quality management system contribution in the form of a standardised sheet (SOP, patient information sheet, questionnaire, etc.)

Attachment 3 to Schüle D, Sonnek B, Lübbert J, Kurcrzyk S, Glassen K, Loukanova S. *From competency mapping to curriculum development: identifying gaps in an undergraduate general practice clerkship – a mixed-methods study*. GMS J Med Educ. 2026;43(6):Doc79. DOI: 10.3205/zma001873

Point in time		Week 1	Week 2	Week 3 & 4	Week 5 % 6	
		**Preclinical training (2nd semester)**	**Preclinical training (4th semester)**	**Beginning of clinical training**	**End of clinical training**	**Over course of longitudinal clerkship**
**CanMEDs**	**NKLM 2.0**					
Professional	VI. Professional conduct, ethics, medical history and law	patient-doctor-relationship, dealing with embarrassment in exam situations	burn-out prevention and self-care	Shared decision making	Error management	
Medical expert	VII. Practical clinical skills	Physical exam	Administering vaccinations, blood draw	Geriatric assessment	Interpreting laboratory results	

Attachment 3 to Schüle D, Sonnek B, Lübbert J, Kurcrzyk S, Glassen K, Loukanova S. *From competency mapping to curriculum development: identifying gaps in an undergraduate general practice clerkship – a mixed-methods study*. GMS J Med Educ. 2026;43(6):Doc79. DOI: 10.3205/zma001873
