# Supplementary material for: From competency mapping to curriculum development: Identifying gaps in an undergraduate general practice clerkship – a mixed-methods study
Source: GMS J Med Educ. 2026 Sep 15;43(6):Doc79. doi: 10.3205/zma001873 (PMC13617322; doi:10.3205/zma001873)
Supplement: Supplementary file 4 [file JME-43-79-s-004.pdf]

Attachment 4: Competency-based logbook for general practice block clerkship

Competency levels/Learning objectives (Target competency level at end of clerkship shown in brackets)	CanMEDs	NKLM 2.0	EPA	Self-assessment of competency level before clerkship	Focus points set together (Student and preceptor)	Self-assessment of competency level after clerkship	Preceptor assessment of competency level
**Evaluation requirements: 10 focus points** must be selected for evaluation, including at least:<br>• 3 SOAP cases<br>• 1 Screening examination<br>• 1 Counselling (on colonoscopy, vaccinations, cardiovascular risk with ARRIBA tool OR lifestyle)<br>• 1 Laboratory findings interpretation							
**Competency level according to NKLM 2.0**<br>**0** = no knowledge<br>**1** = Factual knowledge: Descriptive knowledge (facts, information) - name and describe<br>**2** = Practical knowledge and rationale: Explain facts and contexts, classify in clinical setting<br>**3a** = Practical competency: perform and demonstrate under supervision<br>**3b** = Practical competency: perform independently and situation-appropriately with knowledge of consequences							
**Staying healthy**							
Issuing a health insurance or private prescription (2)	Communicator	II. Professional communication	4.2				
Assessment of work incapacity incl. certificate if applicable (2)	Communicator	II. Professional communication	4.2				
Issuing a therapy or medical aid prescription (2)	Communicator	II. Professional communication	4.2				
Cardiovascular risk counselling using ARRIBA risk score (3a)	Communicator, Health advocate	II. Professional communication,<br>IV. Health counselling, prevention and promotion	3.2				
Performing a screening examination (3a)	Medical expert	VII. Practical clinical skills	1.1, 2				
Creating a vaccination plan (3a)	Health advocate	IV. Health counselling, prevention and promotion	1.1, 1.4, 3.1, 3.2				

Performing a vaccination (3a)	Medical expert	VII. Practical clinical skills	2, 3.1				
Conducting colonoscopy or IFOBT counselling as part of cancer screening (2)	Communi-cator, health advocate	II. Professional communication, IV. Health counselling, prevention and promotion	3.2				
**Getting healthy**							
**Acute care for a patient with one of the following reasons for encounter, based on a guideline, and subsequent case presentation and documentation using the SOAP format (NKLM VIII.1-03.5; VIII.2-06.1.1)**							
Please select ***one*** *of the options:*							
Chest pain (DEGAM* guideline) (3a)	Scholar, communi-cator, medical expert	I. Medical scientific skills, II. Professional communication, VII. Practical clinical skills	1.1, 1.2, 1.3, 1.4, 4.1, 4.2, 4.3, 5.2				
Nonspecific back pain (NVL**) (3a)	Scholar, communi-cator, medical expert	I. Medical scientific skills, II. Professional communication, VII. Practical clinical skills	1.1, 1.2, 1.3, 1.4, 4.1, 4.2, 4.3, 5.2				
Dizziness (DEGAM guideline) (3a)	Scholar, communi-cator, medical expert	I. Medical scientific skills, II. Professional communication, VII. Practical clinical skills	1.1, 1.2, 1.3, 1.4, 4.1, 4.2, 4.3, 5.2				
Fatigue (DEGAM guideline) (3a)	Scholar, communi-cator, medical expert	I. Medical scientific skills, II. Professional communication, VII. Practical clinical skills	1.1, 1.2, 1.3, 1.4, 4.1, 4.2, 4.3, 5.2				
Gout attack (DEGAM guideline) (3a)	Scholar, communi-cator, medical expert	I. Medical scientific skills, II. Professional communication, VII. Practical clinical skills	1.1, 1.2, 1.3, 1.4, 4.1, 4.2, 4.3, 5.2				
Unipolar Depression (NVL) 3a)	Scholar, communi-cator, medical expert	I. Medical scientific skills, II. Professional communication, VII. Practical clinical skills	1.1, 1.2, 1.3, 1.4, 4.1, 4.2, 4.3, 5.2				

**Acute care of a patient with infection based on a DEGAM guideline and subsequent case presentation and documentation using the SOAP format**							
Please select ***one*** *of the options:*							
Cough (3a)	Scholar, communi-cator, medical expert	I. Medical scientific skills, II. Professional communication, VII. Practical clinical skills	1.1, 1.2, 1.3, 1.4, 4.1, 4.2, 4.3, 5.2				
Rhinosinusitis (3a)	Scholar, communi-cator, medical expert	I. Medical scientific skills, II. Professional communication, VII. Practical clinical skills	1.1, 1.2, 1.3, 1.4, 4.1, 4.2, 4.3, 5.2				
Sore throat (3a)	Scholar, communi-cator, medical expert	I. Medical scientific skills, II. Professional communication, VII. Practical clinical skills	1.1, 1.2, 1.3, 1.4, 4.1, 4.2, 4.3, 5.2				
**Living with illness**							
Foot inspection or conducting a DMP*** Diabetes (with pulse status assessment, polyneuropathy diagnostics with tuning fork or monofilament) (3a)	Medical expert	VII. Practical clinical skills	1.1, 2				
Assessing for functional impairments in geriatric patients (e.g., Barthel Index, Timed Up & Go, Clock test, MMST) (3a)	Medical expert	VII. Practical clinical skills	1.1, 2				
Documentation and case presentation using SOAP format: home visit patient (3a)	Communi-cator, medical expert	II. Professional vommunication, VII. Practical clinical skills	1.1, 1.2, 1.3, 1.4, 4.1, 4.2, 4.3				
Medication plan review with interaction check and optimisation (3a)	Health advocate, medical expert	IV. Health counselling, prevention and promotion, VII. Practical clinical skills	1.4, 5.3				
**Additional learning objectives**							
Performing a dressing change, wound care for acute wounds (3a)	Medical expert	VII. Practical clinical skills	2, 3.1				
Treatment of chronic wounds (2)	Medical expert	VII. Practical clinical skills	2, 3.1				

Documentation and case presentation using SOAP format: Interface patient (hospital discharge/admission (3a)	Communi-cator, medical expert	II. Professional communication, VII. Practical clinical skills	1.1, 1.2, 1.3, 1.4, 4.1, 4.2, 4.3				
Documentation and case presentation using SOAP format: Trajectory assessment (Patient seen several times) considering the principle of watchful waiting (3a)	Communi-cator, medical expert	II. Professional communication, VII. Practical clinical skills	1.1, 1.2, 1.3, 1.4, 4.1, 4.2, 4.3				
Laboratory findings interpretation (2)	Medical expert	VII. Practical clinical skills	1.3				
Suture or staple removal (3a)	Medical expert	VII. Practical clinical skills	2				
Post-mortem examination (2)	Pro-fessional, medical expert	VI. Professional conduct, ethics, medical history and law, VII. Practical clinical skills	2, 4.3				
Abdominal ultrasonography (3a)	Medical expert	VII. Practical clinical skills	2				
Thyroid ultrasonography (3a)	Medical expert	VII. Practical clinical skills	2				
Spirometry (2)	Medical expert	VII. Practical clinical skills	2				
Digital-rectal examination (3a)	Medical expert	VII. Practical clinical skills	2				
Evaluation of 24-hour blood pressure monitoring (2)	Medical expert	VII. Practical clinical skills	1.3				
Drawing therapeutic consequences from 24-hour ECG findings (2)	Medical expert	VII. Practical clinical skills	1.3				
Lifestyle counselling (nutrition, exercise, smoking cessation) (3a)	Communi-cator, health advocate	II. Professional communication, IV. Health counselling, prevention and promotion	3.2				
Home visits:<br>- Assessment of tripping hazards (2)<br>- Assessment of patient's ability to take care of themselves (2)	Health advocate, medical expert	IV. Health counselling, prevention and promotion, VII. Practical clinical skills	3.2, 5.3				
Pain management according to WHO step scheme (3a)	Medical expert	VII. Practical clinical skills	1.4				

Nursing home visit (2)	Collabora-tor, medical expert	III. Interprofessional competencies,<br>VII. Practical clinical skills	1.1 1.2 1.3 1.4 4.2				
Blood draw (3b)	Medical expert	VII. Practical clinical skills	2, 3.1				

* DEGAM (Deutsche Gesellschaft für Allgemeinmedizin und Familienmedizin), German Society of General Practice and Family Medicine
** NVL (Nationale Versorgungsleitlinie), National disease management guideline
*** DMP, Disease Management Programme
